# Shift of fleshy fruited species along elevation: temperature, canopy coverage, phylogeny and origin

**DOI:** 10.1038/srep40417

**Published:** 2017-01-13

**Authors:** Shunli Yu, Ofir Katz, Weiwei Fang, Danfeng Li, Weiguo Sang, Canran Liu

**Affiliations:** 1 State Key Laboratory of Vegetation and Environmental Change, Institute of Botany, Chinese Academy of Sciences, China; 2 Department of Geography and Environmental Development, Ben-Gurion University of the Negev, Israel; 3 The Dead Sea and Arava Science Center, Tamar Regional Council 8691000, Israel; 4 College of Life and Environmental Science, Minzu University of China, Beijing, China; 5 Department of Environment, Land, Water and Planning, Arthur Rylah Institute for Environmental Research, Heidelberg, VIC 3084, Australia

## Abstract

Plant communities differ in their fruit type spectra, especially in the proportions of fleshy and non-fleshy fruit types. However, which abiotic and biotic factors drive this variability along elevation gradient and what drives the evolution of fruit type diversity still are puzzling. We analyzed the variations in proportions and richness of fleshy-fruited species and their correlations to various abiotic and biotic variables along elevation gradients in three mountains in the Beijing region, northeast China. Fleshy-fruited species, which are characterized by high fruit water contents, were found in great proportion and richness at relatively low elevations, where soil water content is low compared to high elevations. High temperatures in low elevations increase water availability for plants. Plants that grow in the shaded low-elevation thick-canopy forests are less exposed to evapotranspiration and thus possess water surpluses that can be invested in fleshy fruits. Such an investment in fleshy fruits is beneficial for these species because it makes the fruits more attractive to frugivores that act as seed dispersers in the close-canopied environments, where dispersion by wind is less effective. A hypothesis is proposed that plant internal water surpluses are the prerequisite conditions that permit evolution of fleshy fruits to occur.

In traditional botany, fruits are classified into fleshy fruits and non-fleshy (dry) fruits based on their water content when ripe. This dichotomy reflects a trade off in seed protection mechanisms: pericarp softness in fleshy fruits and lignification in dry fruits^1^. The origins, evolution and diversity of fleshy fruit pericarp have attracted researchers’ attention, but remain somewhat ambiguous^1^,^2^,^3^. Serving a function in protecting, nurturing and dispersing seeds, pericarp coupled with double fertilization played a profound role in the global prevalence of angiosperms^1^.

Nonetheless, fleshy fruit pericarps do not only protect seeds^1^, but also assist in their dispersal^4^,^5^,^6^, being attractive to frugivores that may act as seed dispersers^7^,^8^,^9^. Frugivores usually consume the fleshy pericarps, leaving the seeds mostly intact and unintentionally disperse them through their feces. Species with seeds that are too large to be effectively dispersed by wind (anemochory), and thus require animals as dispersal agents^10^, are characteristic of shaded habitats with low light intensity and potential evapotranspiration^5^, so it is not surprising that fleshy-fruited species also tend to be more common in such habitats^11^. Therefore, fleshy fruit types are thought to have coevolved with endozoochory (seed dispersal through ingestion)^12^. Until now, this possibility has only been studied from the viewpoint of fruit size^11^,^13^. Thus, it was proposed that fleshy fruit pericarp first evolved owing to its advantages in seed protection, whereas its attractiveness to frugivores as seed dispersers was only a secondary advantage^1^, or that fruit type evolution is driven by climate^14^ or vegetation dynamics^11^. The existing theories present a mixture of physiological mechanisms (i.e., conditions that support the development of fleshy fruits) and evolutionary ecology (i.e., what makes fleshy-fruited species beneficial), and it is not always clear to what degree any abiotic and biotic factor contributes to the physiological mechanisms and/or to the evolutionary ecology. Thus, the underlying mechanisms of fruit type ecology and evolution still remain puzzling.

To better understand plant fruit type ecology and evolution, this phenomenon should also be studied in the contexts of plant communities and landscape ecology. Plant communities are characterized, amongst other traits, by their seed dispersal spectra^4^,^15^ and fruit type spectra^16^,^17^,^18^,^19^. Despite being two different concepts, seed dispersal and fruit type spectra probably share a certain degree of similarity and overlap in species composition at the community level, as a result of the inherent association of fruits and seeds (as discussed above). Seed dispersal spectra variations are largely the result of variations in exposure to high wind velocities on the one hand, and of the occurrence and abundance of frugivores that can act as seed dispersers on the other hand^4^,^15^,^20^. Not so differently, fleshy-fruited species proportion and richness are commonly high in low-evapotranspiration shaded habitats^11^, where wind velocities are likely to be low. Moreover, fleshy-fruited species proportion and richness also are high in habitats that are characterized by great amounts of rainfall^21^,^22^,^23^,^24^,^25^. Seeing that fleshy pericarp is in the simplest manner— a water-rich fruit, one may infer that fleshy fruits are common where more water is available to plants. Nevertheless, fleshy-fruited species proportion and richness also are higher in more fertile soils^5^ and forest or woody vegetation^4^,^12^,^15^,^22^,^26^, implying that fleshy-fruited species may be more abundant in shaded understories in which plants are less exposed to direct sunlight and wind. Hence, multiple abiotic and biotic variables are probably responsible for determining fruit type spectra, suggesting that understanding variations in fruit type spectra requires more in-depth look at large environmental gradients that provide heterogeneity of relevant environmental variables, i.e., regional-scale study is advised.

Fruit type spectra variations reflect variations in the relative metabolic costs of constructing and maintaining fleshy fruit pericarps, which depend on both abiotic and biotic characteristics of habitats and plant communities^20^,^27^,^28^. How these multiple abiotic and biotic variables shape regional-scale fruit type spectra variations among plant communities is unclear. Numerous studies have demonstrated how regional-scale elevation gradients, which commonly reflect gradients of multiple abiotic and biotic variables, cause variations in evaporation^29^, soil temperature and moisture^30^, plant community composition and structure^31^, species richness and diversity^23^,^32^,^33^, vegetation distribution^34^, seed mass^35^, leaf size spectra^36^, plant stature^32^ and seed dispersal spectra^4^,^23^. Although previous studies have shown that fleshy-fruited species richness is great at low elevations^16^,^17^,^18^,^37^, the mechanistic causes of this trend still remain unclear.

Temperature, precipitation, soil moisture and soil nutrient contents interactively affect species richness and distribution along elevation gradients, with soil moisture being more significantly effective in low elevations whereas temperature being more significant in high elevations^30^. This may be attributed in part to evaporation from the soil surface generally decreasing as elevation increases^29^. In the present study, we investigated proportion and richness of fleshy-fruited species in various plant communities of various vegetation formations (shrub communities, alpine meadows, artificial forests and natural forests) along elevation gradients. By observing abiotic traits (measured soil and air temperature and soil moisture) and biotic species and community traits (documented vegetation formation and plant habits, and putative seed dispersers deduced from the literature) in each habitat, we discuss how abiotic and biotic variables shape regional-scale fruit type spectra variations among plant communities. Acknowledging that fruit types tend to be phylogenetically conservative at the family level, we also taken into account the effect of phylogenetic signal or diversity on the pattern of fleshy fruited species distribution in our analysis.

## Results

### Variation of fleshy-fruited species proportion and richness along elevation gradients

**Figure 1:**
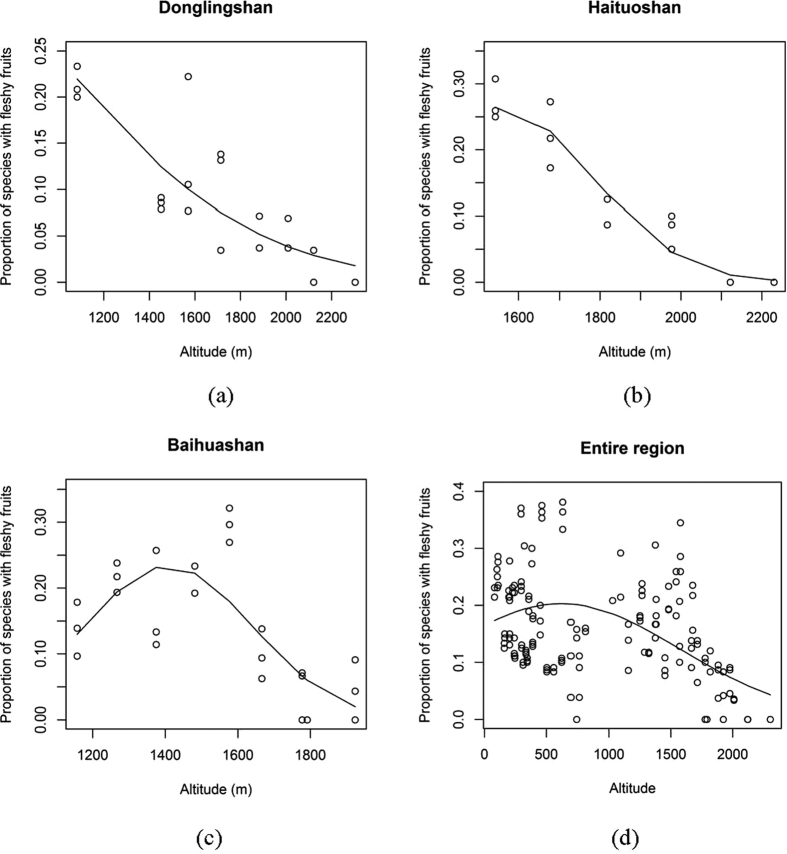
Relationships between elevation and proportions of species with fleshy fruits for each mountain separately ((**a**) Donglingshan, (**b**) Haituoshan, (**c**) Baihuashan) and for the entire study area (**d**). Solid lines represent the best fit model for each mountain.

Fleshy fruited species proportions varied greatly across communities, from 0% to 45.0%, in all three studied mountains and in the entire region (Fig. 1). Herbaceous communities had lower fleshy-fruited species proportions than woody communities (0–12.5% and 3.5–45%, respectively). Fleshy-fruited species proportions in artificial forests (with introduced species, including *Larix principis-rupprehtii* and *Platycladus orientale*) did not differ from those found in natural forests. Fleshy-fruited species proportion declined significantly with elevation (p < 0.05) along the entire gradients in Donglingshan and Haituoshan (Fig. 1a,b). In Baihuashan, fleshy-fruited species proportion was greatest at approximately 1577 m elevation (Fig. 1c), declining both above and below this elevation, and reaching a minimum at the summit. For the entire region, including communities at lower elevations between the three mountains, the trend of decreasing proportion of fleshy-fruited species also was apparent at elevations above 600 m (Fig. 1d).

For all three mountains, the best fit models (Table 1) suggested that elevation had a significantly negative effect on fleshy-fruited species proportion, regardless of whether elevation itself or its quadratic transformation was used. Models that include community attributes (number of species, genera and families) were mostly rejected, and even in the few cases that such models were selected, the effects of community attributes were insignificant. The phylogenetic index PD was not included in any best fit model (Table A1). Notably, for Baihuashan and the entire regions, the best fit models also included NF and PD; however, in Baihuashan these effects were statistically insignificant, and in the entire region NG and PD had much less significant effects than elevation had (Table 1).

Regression analysis showed that fleshy-fruited species richness of each growth form separately displayed a similar decrease with elevation (Table 2). In Donglingshan, fleshy-fruited species richness of each growth form declined with elevation, and the trend was observed only for woody species in Haituoshan. In Baihuashan, the highest fleshy-fruited species richness occurred at 1577 m elevation.

### Fruit water content

Fleshy fruits indeed had higher water content than dry fruits when ripe. Berries (including pomes, pepos, hips, aggregate fruits and polythalamic fruits) had the highest average water content (72.82%), followed by drupes (62.41%) and nuts (35.11%), while water content of dry fruits was generally below 40% (Table A2). Therefore, the decreasing proportion or fleshy-fruited species richness with elevation represents a decline in ripe fruit water content with elevation.

## Discussion

### Variation of fleshy-fruited species proportion and richness along elevation gradients: temperature and shaded habitats

**Figure 2:**
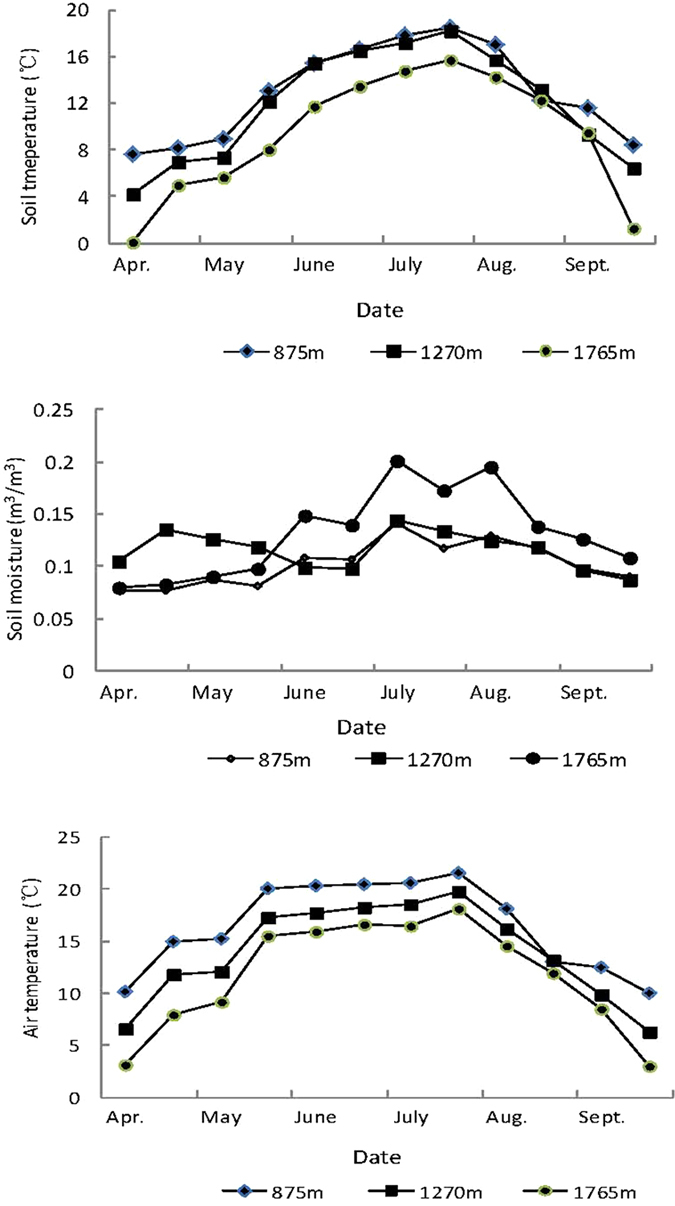
Soil temperature (**a**) and moisture (**b**) at 5 cm and 10 cm below ground as well as air temperature (**c**) (measured by HOBO weather station data recorder, Onset Company, USA) from April to September 2011. Similar patterns were observed in 2012.

Although simple logic would suggest that water-rich fleshy-fruited species are to be more common where soil moisture is greater, our results clearly demonstrate the opposite (Figs 1 and 2). This disparity cannot be dismissed by shifts in dominant plant growth form or phylogenetic groups along the elevation gradients (Tables 1 and 3). Thus, it is unlikely that fleshy-fruited species proportion variations are simply determined by soil moisture, but other environmental variables. The combination of low temperatures^4^,^20^,^23^ and low density or absence of tree canopies^38^,^39^,^40^ at high elevations are unfavorable conditions for fleshy-fruited species to grow in, because of the increased risk of physiological drought^41^,^42^ that is probably even more severe to plants that invest large amounts of water in their fruits. Therefore, the cool conditions at high elevations limit plants’ ability to take up water and invest it in fleshy fruits with high water content, decreasing fleshy-fruited species frequency or richness at high elevation^4^,^11^,^15^,^20^,^22^,^25^. Hence, especially at higher elevation, temperature plays a more important role than soil moisture in controlling the presence or absence of fleshy fruited species.

Further support to the role that canopy density and evapotranspiration play in controlling fruit type spectra variations can be found in the case of Baihuahsan, the mountain in which fleshy-fruited species were most common at mid-elevations (1577 m) rather than monotonously declining with elevation (Fig. 1c). According to our observations, in Donglingshan and Haituoshan forest canopy coverages decline with increasing elevation. However, in Baihuashan, slope grades are moderate (10°) at elevations of approximately 1577 m, compared to slope grades at low and high elevations (20° and 25°, respectively). As a result, canopy cover also is high at moderate elevation (90%) compared to low and high elevations (70% and 75%, respectively). Mid-elevations in Baihuashan are thus more shaded, and these conditions are more favorable for the survival of fleshy-fruited species than at high and low elevations.

Lastly, favorable seed dispersal strategies also differ between high and low elevations. In high elevation wind-blown open meadows, anemochory is an effective seed dispersal strategy, so the metabolic costs of attracting frugivores as seed dispersers make endozoochory an inferior seed dispersal strategy (possibly even disadvantageous). On the other hand, in the shaded and closed low elevation forests anemochory is not an effective seed dispersal strategy due to the much reduced wind speeds, physical obstacles that the dense vegetation creates and relatively low abundance of vacant space in which seedlings can establish. Therefore, plants that invest water in fleshy fruit pericarps and thus attract frugivores as seed dispersers are more advantageous at low elevations than at high elevations. In the Beijing region, bird and mammal species richness are greater in forests (108 and 28 species, respectively) compared to meadows (15 and 13 species, respectively)^43^, as is the case in other parts of the world as well^44^,^45^. Therefore, frugivores as seed dispersers are probably more abundant at low elevations, so anemochory is the dominant seed dispersal strategy in wind-blown high elevation meadows while endozoochory is the dominant dispersal strategy in shaded forests of low elevation^4^,^23^,^45^. This dichotomy is further strengthened by short-lived herbaceous species growing in open or unstable habitats typically producing large amounts of relatively small propagules that are easily dispersed by wind^46^,^47^,^48^. Nonetheless, frugivore occurrence and distribution in itself relies on fleshy fruits being abundant, and for fleshy fruits to be able to exist certain abiotic conditions need to exist (as discussed above). Therefore, any effects of biotic plant-frugivore interactions on fleshy-fruited species abundance are only secondary to other factors.

### Possible extrapolations of long-term evolution for fleshy species

While inter-specific fruit type variations are thought to be related to plant phylogeny, especially above the species level^25^, phylogenetic signal was not found to have a significant effect on fleshy-fruited species proportions in this study (Tables 1, 3 and A1). Nevertheless, species that are common at low elevations but are absent at high elevations in the study region belong to multiple plant families across the entire angiosperm phylogenetic tree (Table A1). Hence, the small effect of phylogenetic signal or diversity among fleshy-fruited species on their proportion along elevation gradients may reflect phylogenetic conservatism of fruit types at the family level and a large phylogenetic variety of families in all plant communities.

We acknowledge that, to a certain extent, fleshy-fruited species may adapt to the changing environment by decreasing fruit size and fruit water content or by increasing relative fruit protein and sugar contents to alleviate drought and freezing stresses^10^. However, species elasticity, adaptive amplitudes and variability are sometimes limited, so such species are not expected to exist above an elevation at which temperature may be too low to allow plant survival. Therefore, we suggest that the little effect of phylogeny on fleshy-fruited species proportions results from long-term qualitative changes of plant traits (e.g., fruit type and water content) as angiosperms have evolved, radiated and adapted to environmental variability.

In the Beijing region, fleshy-fruited species are considerably more common among lianas (52.2%), shrubs (50.8%) and trees (37.2%) than among herbaceous species (6.4%)^13^. More specifically, we suggest that woody species (1) have affinity to low elevation, (2) prefer shaded habitats, (3) are physiologically able to take up more water through their extensive root systems and strong vascular systems, and (4) compensate for water deficiency during short droughts and dry seasons by accumulating water surpluses during mild periods. Therefore, they are capable of allocating more water resources to produce high-water content fruits compared to more short-lived herbaceous species. The final result is woody species more abundantly having fleshy fruits^13^,^26^, which is an example for parallel evolution of vegetative and reproductive traits in plants facing similar climatic changes^14^,^49^. Hence, our results and their interpretation reflect the possible evolution of fleshy-fruited species in warm and shaded habitats.

Finally, our results imply that dry- and fleshy-fruited species reflect adaptations to different types of abiotic and biotic conditions, which do not negate each other. Dry-fruited species are better adapted to physiological droughts, while fleshy-fruited species are better adapted to low anemochory potential; so, both fruit types coexist in many habitats around the world, albeit at different proportions. Nevertheless, low anemochory potential is frequently associated with reduced physiological droughts, due to the shared effect of shading and the extensive vascular systems of woody species (trees, shrubs and lianas). Therefore, the greater proportions of dry-fruited species in all studied communities (Fig. 1) can be explained by dry fruits being less resource-demanding and less dependent on endozoochory and thus adapted to a broader range of ecosystems than fleshy fruits are. Furthermore, it is more reasonable that early successional stages (e.g., meadows), which are exposed to strong wind and evapotranspiration, would be dominated by dry-fruited species. The first fleshy-fruited species to establish in a community are probably woody species, owing to their extensive vascular systems that provide water surpluses. Only in later successional stages, when woody species provide sufficient shading and reduce evapotranspiration and wind, can fleshy-fruited species significantly increase in proportions, but never or very rarely fully suppressing dry-fruited species.

Dry fruited species predominance in various community types may result from the early origins of angiosperms in the cold and dry climates of the Late Jurassic and Early Cretaceous^50^,^51^. Fleshy fruited species in Beijing belong to multiple plant families, including both primitive (such as Juglandaceae) and derived lineages (such as Liliaceae) (Table A2), indicating continual speciation throughout the evolutionary history of this region^49^. Fleshy fruits probably did not become prolific until the Late Cretaceous, when climate became warm and frugivorous birds and mammals first emerged^52^. The proliferation of fleshy-fruited species today depends largely on vegetation structure^11^ and climatic conditions^14^, suggesting that fleshy fruitedness may be a derived trait that may have evolved as climate conditions ameliorated and became warmer and more humid during the Mid-Cretaceous^51^. Our results and the results of previous studies suggest that the origins of this trait should probably be sought in tropical regions, or in woody species growing at low elevations in shaded habitats of temperate regions.

## Conclusions

Fleshy-fruited species proportion and richness decrease with increasing elevation in the Beijing region. This pattern can be primarily explained by low elevations being characterized by high temperatures and closed-canopy woody plant communities. Under such conditions, plants are more likely to acquire ample water amounts, providing them with surpluses that can be invested in fleshy fruit pericarps with high water content. In addition, the closed canopy limits anemochoric seed dispersal, so fleshy fruits have the added advantage of attracting frugivore that act as seed dispersal agent. The strong association of fleshy-fruited species with warm and shaded habitats, alongside the phylogenetic conservatism of fruit types within families and richness of fleshy-fruited families across the angiosperm phylogenetic tree, suggests that fleshy-fruited species have probably evolved in such habitats. Hence, seed dispersal and fruit type spectra variations are likely to reflect the ecology and evolution of plant resource acquisition and allocation strategies in different habitats. Therefore, fruit type spectra differences are the result of multiple interacting environmental variables, implying that the ecology and evolution of fruits may be more complex than commonly viewed. Further studies of fruit type spectra along large-scale environmental gradients will surely contribute to our understanding of fruit type diversity mechanisms and their evolution.

## Methods

### Site description and sampling

The mountainous part of the Beijing region (39°26′–41°03′N, 115°25′–117°30′E) has a warm temperate climate. Annual mean rainfall is 525 mm, with rains occurring mostly from March to September. In general, soil and air temperatures decrease with elevation, but soil moisture exhibits a contrasting pattern (Fig. 2). The prevalent plant communities are deciduous broad-leaved forests (such as *Quercus wutaishanica* community, *Quercus variabilis* community, *Juglans mandshurica* community, mixed broad-leaved forest, *Betula platyphylla* community), coniferous forests (such as *Pinus tabulaeformis* community, *Larix principis-rupprehtii* community and *Platycladus orientale* community), shrub communities (such as *Vitex nengudo* var. *heterophylla* community, *Syringa pekinensis* community) and meadows (alpine meadow and *Themeda japonica* community). These prevalent plant communities were sampled from 2011 to 2013. We have attempted, as much as possible, to study only mature communities that have not been disturbed by humans or livestock in recent years.

Elevation gradients were studied on three mountains, Donglingshan, Baihuashan and Haituoshan, less than 100 km apart from each other. On each mountain, plant communities were sampled at elevation intervals of approximately 100 m, along transects (on the same slope aspect) from elevations of 800 m (broad-leaved forests) to as much as 2300 m (deciduous shrub communities or alpine meadows). At each elevation, we have set one plot (at least 3 community quadrats per plot) in each of the prevalent plant communities found. The total number of plots was 8 on Donglingshan, 9 on Baihuashan and 6 on Haituoshan. In addition, 40 plots were set at lower elevations (100–1100 m) in the area between the three mountains. In forest communities, three 10 × 10 m quadrats were randomly sampled in undisturbed areas (or mildly disturbed areas). In shrub and herbaceous communities, three 5 × 5 m and at least five 1 × 1 m quadrats were sampled, respectively (sufficient area to represent each community type). All indigenous species were recorded in each quadrat. Alien species were rare, and were assumed to have little effect on community composition or structure. The geographical coordinates and setting (latitude, longitude, elevation and slope aspect) were measured using wireless GPS logger (HOLUX Technology Inc., Taiwan). Other physical characters of the plots, such as canopy coverage, grade of slope and micro-topography were recorded.

### Fruit water content and fruit type classification

Ripe fruits were collected from at least 3 individuals of the most common species, and their fresh weight was measured as soon as possible. Fruits were then allowed to air-dry to a constant mass in the laboratory before dry biomass was weighed. Depending on the size and number of fruits per individual, measurements were averaged for 3–30 fruits per species.

Fruits were classified as fleshy if they were described in the flora as berries, drupes, pomes, rose hips, multiple fruits and pepos or as possessing fleshy pericarp or succulent tissue in general (including arils). Accordingly, capsules, achenes, nuts, caryopses, legumes, follicles, pods, cremocarps, utricles, samaras and schizocarps were classified as non-fleshy (dry) fruits. Some species (e.g., *Cotinus coggygria* var*. cinerea* and *Vitex nengudo* var*. heterophylla*) that were considered to be fleshy-fruited based on fruit morphology were reclassified as dry-fruited owing to very low water content (Table A2).

### Statistical analysis

A phylogenetic tree was constructed for all the seed plants in the communities studied using Phylomatic (version 3) with an APG III derived mega-tree as the base tree^52^,^53^,^54^. Bladj block in the software Phylocom (version 4.2, available online: http://phylodiversity.net/phylocom/) was used to assign branch lengths to the obtained tree. For each quadrat, we calculated the phylogenetic index PD, the sum of unique branch lengths of species in the quadrat, which served as a measure of phylogenetic diversity.

In order to characterize the relationships between the proportion of fleshy-fruited species in a community and elevation, generalized linear models (GLMs) with Poisson family and log link were fitted, and the total number of species in each individual quadrat was taken as an offset. For each plant community, the following attributes were considered: elevation, number of families (NF), number of genera (NG) and PD (the latter three corrected against total species number). Since these attributes were significantly correlated (r > 0.68, p < 0.05, n = 66), we tested models that included no more than two attributes. In addition to the linear term of elevation, we also considered the square of this variable. In total, 12 models were constructed (Table 1) and evaluated for each mountain separately and for the entire region. We selected the model with the lowest AICc value and any other model with ∆AICc < 2. Additionally, regression models were used to analyze whether species richness of various growth forms and the whole plants with fleshy fruits were related to elevation, respectively.

All the analyses were implemented in R3.1.1^55^. GLMs were built with the function glm within R package stats, and model selection was made with the function aictab within R package AICcmodavg.

## Additional Information

**How to cite this article**: Yu, S. *et al*. Shift of fleshy fruited species along elevation: temperature, canopy coverage, phylogeny and origin. *Sci. Rep.* **7**, 40417; doi: 10.1038/srep40417 (2017).

**Publisher's note:** Springer Nature remains neutral with regard to jurisdictional claims in published maps and institutional affiliations.

## Supplementary Material

Supplementary Appendixes Tables

## Figures and Tables

**Table 1:** Selected models for the three mountains and pooled data.

	Intercept	Elevation	Elevation^2^	NG	PD
Donglingshan					
md12	−0.8029**		−6.080 × 10^(−7)^***		
md8	0.5174 ns	−0.0018***			
Haituoshan					
Md4	−25.26 ns	0.0031*	−9.901 × 10^(−6)^*		
Md12	1.71**	−1.206 × 10^(−6)^***			
Md8	5.3871***	−0.0042***			
Baihuashan					
md4	−19.86***	−0.0261***	−9.281 × 10^(−6)^***		
md3	−20.36***	0.0290***	−1.032 × 10^(−5)^***		−2.521 × 10^(−4)^ns
md2	−20.18***	−0.0279***	−9.919 × 10^(−6)^***	−0.033 ns	
Entire region					
md2	−9.821***	0.0135***	−4.949 × 10^(−6)^***	−0.0350**	
md3	−9.377***	0.0133***	−4.909 × 10^(−6)^***		−2.112 × 10^(−4)^**

Note: ***p < 0.01; **p < 0.05; *p < 0.1; ns*p* ≥ 0.1.

**Table 2:** Results of regression analysis on species richness of different growth forms with fleshy fruits along altitudinal gradient.

Sites	Growth forms	F values	P values
Donglingshan mountain	Woody species	21.638	0.003
	Herb	18.700	0.005
	Liana	8.149	0.029
Haituoshan mountain	Woody species	37.882	0.009
	Herb	7.972	0.067
	Liana	1.530	0.304
Baihuashan mountain	Woody species	2.965	0.129
	Herb	1.799	0.222
	Liana	1.683	0.236

**Table 3:** Variables included in each model.

Model	Elevation	Elevation^2^	NF	NG	PD
md1	yes	yes	yes		
md2	yes	yes		yes	
md3	yes	yes			yes
md4	yes	yes			
md5	yes		yes		
md6	yes			yes	
md7	yes				yes
md8	yes				
md9		yes	yes		
md10		yes		yes	
md11		yes			yes
md12		yes			
