# Supplementary material for: Shift of fleshy fruited species along elevation: temperature, canopy coverage, phylogeny and origin
Source: Sci Rep. 2017 Jan 13;7:40417. doi: 10.1038/srep40417 (PMC5233997; doi:10.1038/srep40417)
Supplement: Supplementary Appendixes Tables [file srep40417-s1.pdf]

Shift of fleshy fruited species along elevation: temperature, canopy coverage, phylogeny and origin

Shunli Yu[1,]*, Ofir Katz[2], Weiwei Fang[1], Danfeng Li[1], Weiguo Sang[3,1] and Canran Liu[4]

[1] State Key Laboratory of Vegetation and Environmental Change, Institute of Botany, Chinese Academy of Sciences, China

[2] Department of Geography and Environmental Development, Ben-Gurion University of the Negev, Israel

[3] College of Life and Environmental Science, Minzu University of China, Beijing, China

[4] Department of Environment, Land, Water and Planning, Arthur Rylah Institute for Environmental Research, Heidelberg, VIC 3084, Australia

Appendixes

Table A1

Results of model selection for the three mountains and the entire region

Donglingshan			Haituoshan			Baihuashan			Entire region		
Model	AICc	Delta_AICc	Model	AICc	Delta_AICc	Model	AICc	Delta_AICc	Model	AICc	Delta_AICc

md12	68.35	0	md4	50.88	0	md4	104.92	0	md2	231.43	0
md8	69.52	1.18	md12	51.22	0.34	md3	105.6	0.68	md3	232.01	0.58
md4	70.39	2.04	md8	52.28	1.4	md2	106.16	1.24	md4	233.75	2.32
md10	70.66	2.32	md1	54.08	3.2	md1	107.29	2.37	md1	235.31	3.88
md9	70.95	2.61	md11	54.12	3.25	md12	117.67	12.75	md12	251.71	20.28
md11	71.04	2.7	md9	54.13	3.25	md8	119.53	14.61	md10	253.69	22.26
md6	71.28	2.94	md10	54.13	3.25	md9	120	15.08	md11	253.69	22.26
md5	71.89	3.54	md2	54.23	3.35	md10	120.01	15.09	md9	253.89	22.45
md7	72.1	3.75	md3	54.23	3.36	md11	120.14	15.22	md8	258.91	27.48
md3	73.25	4.9	md6	55.18	4.31	md6	121.99	17.07	md5	260.94	29.51
md2	73.39	5.04	md7	55.19	4.32	md5	122.05	17.13	md7	261.1	29.67
md1	73.4	5.06	md5	55.2	4.32	md7	122.08	17.16	md6	261.1	29.67

Table A2 Water content of ripe fruits of 77 fleshy fruited species and 5 species with nut in the sampling site of Beijing mountainous area

Family	Fruit type	Species	Water content (%)
Liliaceae	Berry	*Smilacina japonica*	65.81 ± 1.21
	Berry	*Maianthemum bifolium*	82.94 ± 1.14
	Berry	*Polygonatum macropodium*	60.94 ± 2.22
	Berry	*Polygonatum involucratum*	64.22 ± 10.26
	Berry	*Disporum sessile*	83.73 ± 3.62
	Berry	*Smilax stans*	63.89 ± 2.41
	Berry	*Ophiopogen japonicus*	69.44 ± 1.68
	Berry	*Convallaria pillosa*	65.10 ± 1.39
	Berry	*Paris verticillata*	74.11 ± 4.01
	Berry	*Asparagus dauricus*	13.56 ± 3.87
	Berry	*Asparagus trichophyllus*	34.68 ± 6.14
	Berry	*Asparagus schoberioides*	55.75 ± 1.31
Araceae	Berry	*Pinellia ternata*	69.97 ± 1.23
	Berry	*Arisaema heterophyllum*	50.62 ± 1.70
	Berry	*Arisaema amurense*	57.83 ± 0.44
Cucurbitaceae	Berry (Pepo)	*Thladiantha dubia*	89.57 ± 1.00
Caprifoliaceae	Berry	*Lonicera chrysantha*	75.42 ± 1.52

	Berry	*Lonicera chrysantha* var. *longipes*	75.55 ± 2.67
	Berry	*Lonicera microphylla*	87.62 ± 0.55
	Berry	*Viburnum mongolicum*	66.41 ± 1.84
	Berry	*Sambucus sieboldiana*	67.06 ± 2.16
	Berry	*Viburnum sargentii*	84.34 ± 2.14
Rubiaceae	Berry	*Rubia cordifolia*	69.78 ± 1.94
	Berry	*Rubia chinensis*	66.67 ± 0.00
	Berry	*Paederia scandens*	70.18 ± 3.04
Solanaceae	Berry	*Solanum japoensis*	84.14 ± 1.19
	Berry	*Solanum nigrum*	84.16 ± 1.20
	Berry	*Physalis alkekengi*	82.12 ± 0.71
	Berry	*Physalis minima*	86.83 ± 0.28
	Berry	*Physalis angulata*	87.64 ± 1.11
	Berry	*Lysium barbarum*	84.20 ± 2.86
Verbenaceae	Drupe	*Vitex nengudo* var. *heterophylla*	7.87 ± 6.85
Oleaceae	Drupe	*Chionanthus retusus*	67.78 ± 0.19
Ebenaceae	Berry	*Diospyros lotus*	71.29 ± 10.30
Cornaceae	Drupe	*Cornus walteri*	76.56±1.89
	Drupe	*Cornus bretschneideri*	75.94 ± 1.10
Araliaceae	Berry	*Aralia elata*	80.49 ± 0.90
	Berry	*Acanthopanax senticosus*	68.50 ± 4.12

	Berry	*Acanthopanax sessiliflorus*	83.40 ± 2.01
Thymelaceae	Drupe	*Wikstroemia chamaedaphne*	55.32 ± 4.69
Tiliaceae	Drupe	*Grewia biloba*	50.84 ± 6.12
Vitaceae	Berry	*Vitis amurensis*	84.05 ± 3.90
Rhamnaceae	Drupe	*Rhamnus schneideri*	63.88 ± 4.01
	Drupe	*Rhamnus globosa*	70.15 ± 1.45
	Drupe	*Rhamnus davrica*	60.79 ± 3.29
	Drupe	*Sageretia paucicostata*	81.40 ± 4.55
Aracardiaceae	Drupe	*Cotinus coggygria* var. *cinerea*	6.03 ± 1.07
Rosaceae	Pome	*Malus spectabilis*	74.33 ± 3.48
	Pome	*Malus batata*	64.36 ± 1.77
	Drupe	*Amygdalus triloba*	61.02 ± 1.19
	Drupe	*Prunus salicina*	83.59 ± 0.07
	Drupe	*Prunus glandulosa*	82.94 ± 1.57
	Drupe	*Prunus armenica* var. *ansu*	78.04 ± 2.09
	Drupe	*Prunus japonica*	81.11 ± 0.87
	Drupe	*Prunus padus*	63.06 ± 0.44
	Drupe	*Prunus tomentosa*	77.42 ± 1.03
	Drupe	*Prunus dictyoneura*	65.68 ± 0.091
	Drupe	*Prunus davidiana*	70.23 ± 6.79
	aggregate fruit	*Rubus saxatilis*	92.03 ± 0.97

	aggregate fruit	*Rubus craetifolius*	84.74 ± 2.16
	Hip	*Rosa bella*	73.50 ± 2.40
	Pome	*Pyrus ussuriensis*	75.18 ± 5.58
	Drupe	*Sorbus pauhuashanensis*	77.63 ± 3.92
	Drupe	*Crataegus cuneatae*	66.11 ± 3.39
	Drupe	*Crataegus kansuensis*	75.38 ± 3.68
	Drupe	*Cotonoaster multiflorus*	77.00 ± 1.04
	Drupe	*Cotonoaster acutifolius*	61.86 ± 3.95
Saxifragaceae	Berry	*Ribes burejense*	83.56 ± 1.61
Schisandraceae	Berry	*Schisandra chinensis*	78.15 ± 0.68
Berberidaceae	Berry	*Berberis amurensis*	68.88 ± 1.11
	Berry	*Berberis poiretii*	64.16 ± 1.38
Moraceae	multiple fruit	*Morus alba*	88.39 ± 0.70
	multiple fruit	*Morus mongolica*	80.00 ± 3.17
	multiple fruit	*Brousonetia papyrifera*	78.94 ± 2.18
Ulmaceae	Drupe	*Celtis bungeana*	15.13 ± 7.70
	Drupe	*Celtis koraiensis*	59.01 ± 0.48
Juglandaceae	Drupe	*Juglans mandshurica*	65.68 ± 9.34
Fagaceae	Nut	*Quercus acutisima*	40.67 ± 1.31
	Nut	*Quercus variabilis*	38.97 ± 1.61
	Nut	*Quercus wuaishanica*	34.43 ± 7.41

	Nut	*Quercus aliena* var.*acuteserrata*	34.94 ± 1.34
	Nut	*Quercus aliena*	26.53 ± 8.05
